# Different resting membrane potentials in posterior parietal cortex and prefrontal cortex in the view of recurrent synaptic strengths and neural network dynamics

**DOI:** 10.3389/fncel.2023.1153970

**Published:** 2023-07-13

**Authors:** Minsu Yoo, Yoon-Sil Yang, Jong-Cheol Rah, Joon Ho Choi

**Affiliations:** ^1^Korea Brain Research Institute, Daegu, Republic of Korea; ^2^Daegu Gyeongbuk Institute of Science and Technology, Daegu, Republic of Korea

**Keywords:** membrane potential, PFC, PPC, working-memory, network dynamics

## Abstract

In this study, we introduce the importance of elevated membrane potentials (MPs) in the prefrontal cortex (PFC) compared to that in the posterior parietal cortex (PPC), based on new observations of different MP levels in these areas. Through experimental data and spiking neural network modeling, we investigated a possible mechanism of the elevated membrane potential in the PFC and how these physiological differences affect neural network dynamics and cognitive functions in the PPC and PFC. Our findings indicate that NMDA receptors may be a main contributor to the elevated MP in the PFC region and highlight the potential of using a modeling toolkit to investigate the means by which changes in synaptic properties can affect neural dynamics and potentiate desirable cognitive functions through population activities in the corresponding brain regions.

## 1. Introduction

Both the prefrontal cortex (PFC) and posterior parietal cortex (PPC) are critically involved in working memory and decision-making, but their network dynamics differ significantly. For example, in a decision-making task, the PPC encodes evidence with gradually increasing population firing rates, while neurons in the PFC represent them in a categorical manner, showing relatively more abrupt increases in firing rates than the PPC (Hanks et al., 2015). These distinctions in neural network dynamics may give rise to advantageous cognitive functions that compensate for each other in a multiregional manner by forming feedback and feedforward connections between the PFC and PPC (Murray et al., 2017). According to that theoretical study, the relatively gradual increase in the population firing rate in PPC enables the neural network to have less error rate in making decisions, but it is easier to be distracted by another type of choice during the working memory duration. However, the neural activities in the PFC tend to be more rigorous against distractors. These tendencies have been observed in several animal studies (di Pellegrino and Wise, 1993; Constantinidis and Steinmetz, 1996; Qi et al., 2010). Moreover, the main cause of this difference is purportedly the more strongly connected recurrent synapses in the PFC than in the PPC (Murray et al., 2017).

In our experiment, resting membrane potentials (RMPs) in the PFC were significantly higher (∼10 mV) than those in the PPC. Although the study by Murray et al. (2017) showed that recurrent synaptic structures are the main cause of the distinctive neuronal dynamics, the rate-model used in the study is not capable of applying the different levels of RMPs that we observed in our experiment. Therefore, in this study, we used a spiking neural network introduced in a previous study to investigate how different membrane potential levels affect the neural network dynamics of the PPC and PFC, eliciting cognitive functions in these areas (Wang, 2002). One of the most important features of the spiking neural network (SNN) model is that it considers three major synaptic inputs with different time scales to neurons in the network. This helps us understand the type of synaptic inputs that contribute the most to lifted RMPs.

In addition, we compared the effectiveness of the two parameters, recurrent synaptic strength and RMPs. Our results indicate that RMPs are necessary to be elevated for neural networks to behave more uniquely for the necessary cognitive functions: slow ramping up in the PPC and faster in the PFC. Finally, we suggest another possible factor for the lifted RMPs based on the fact that the PFC has stronger recurrent synapses. Neurons in the neural network continue firing, and the recorded neurons may have continuous random inputs from other neurons in the network. By simulating this situation, we show that NMDA synaptic inputs varied by different recurrent synaptic strengths, which may be the main cause for the different membrane potential levels in the PPC and PFC areas.

## 2. Materials and methods

### 2.1. Animals

All animal experiments were conducted with approval from the Animal Experiment Ethics Committee (approval no. IACUC-20-00007) of the Korea Brain Research Institute (KBRI). All experiments were performed using male C57BL/6N mice.

### 2.2. Brain slice preparation

Brain slices were obtained from 7 to 9-week-old male C57BL/6N mice. Animal care and treatment protocols were approved by the Animal Care and Use Committee of the KBRI. After decapitating the mice, their brains were quickly removed and placed in an ice-cold cutting solution of the following composition (in mM): choline chloride, 110; KCl, 2.5; *NaHCO*_3_, 25; *NaH*_2_*PO*_4_, 1.25; glucose, 25; *CaCl*_2_, 0.5; *MgCl*_2_⋅6*H*_2_*O*, 7; sodium ascorbic acid, 11.6; and pyruvic acid, 3. Coronal slices (300 μm) were prepared using a vibratome (VT1200S, Leica, Wetzlar, Germany) and were incubated at 32°C for at least 30 min. During the preparation of acute slices, the solution was oxygenated with 95% *O*_2_ and 5% *CO*_2_.

### 2.3. Electrophysiological recordings

Acute slices of the PPC (−2.0 mm AP, ± 1.5 mm ML from bregma, −0.5 mm DV from the brain surface) and PFC (+1.0 mm AP, ± 0.5 mm ML from bregma, −0.5 mm DV from the brain surface) of mice were placed in a recording chamber filled with a continuous flow of carbogen-saturated (95% *O*_2_, 5% *CO*_2_) aCSF containing the following (in mM): 119 NaCl, 2.5 KCl, 26 *NaHCO*_3_, 1.25 NaH2PO4, 20 glucose, 2 *CaCl*_2_, 1 *MgSO*_4_, 0.4 ascorbic acid, and 2 pyruvic acid for whole-cell patch-clamp recordings. L5 pyramidal neurons of the slices were visualized using infrared differential interference contrast (IR-DIC) microscopy with a BX51WI upright microscope (Olympus, Japan) and a water-immersed 40 objective lens (numerical aperture 0.8). Patch electrodes with 3–5 *M*Ω tip resistances were prepared using a pipette puller (Shutter Instrument, USA) and filled with an internal solution containing (in mM) 20 KCl, 125 K-gluconate, 10 HEPES, 4 NaCl, 0.5 EGTA, 4 ATP, 0.3 TrisGTP, and 10 phosphocreatine (pH 7.2, ∼290–300 mOsm). None of the patched neurons showed spontaneous action potentials, and RMPs were determined by averaging a 100 *ms* recording period at 0 pA in current clamp mode. The initial 100 *ms* of recording data were acquired prior to the application of current clamping in the experiment, allowing us to measure the resting membrane potential before introducing any artificial current through whole cell recording techniques. In order to differentiate between pyramidal cells and interneuron cells, we employed two key criteria: their responses to current injection and the shapes of their action potentials. Notably, when current was injected, the fast-spiking neurons (interneurons) exhibited a greater frequency of firing action potentials compared to the regular-spiking neurons (pyramidal neurons). Additionally, the two types of neurons displayed discernible differences in the shapes of their action potentials. Each type possessed a characteristic waveform pattern that set them apart. The fast-spiking interneurons displayed action potentials with shorter durations and sharper peaks, while the regular-spiking pyramidal neurons exhibited action potentials with longer durations and more gradual peaks, indicative of their role in sustained neuronal activity and the coordination of complex neural processes.

### 2.4. Data analysis and statistics

Data collection and analysis were performed using Clampfit 10.4 (Axon Instruments, Foster City, CA) and AxoGraph X (AxoGraph, Canberra, Australia). Additional data analysis was performed using MATLAB (Mathworks, Natick, MA, USA) or MS Excel (Microsoft, Redmond, WA) to determine statistical significance. The differences in RMP levels were tested using a *t*-test, and the results were considered significant for *p*-values less than 0.05. *P*-values less than 0.01 or 0.001 were indicated on data plots. Error bars in all figures indicate standard errors unless otherwise noted.

### 2.5. Cortical network model

In this study, we employed a recurrent network model, as previously described. For further information, please refer to the original manuscript (Wang, 2002). The neural network simulation code is also publicly accessible, as reported by Gerstner et al. (2014). Here is a brief overview of the model we used.

The model was introduced by Wang (2002) was devised by Amit and Brunel (1997) and Wang (1999), which represents a local circuit in the posterior parietal and prefrontal cortices (Amit and Brunel, 1997; Wang, 1999). It has N neurons, 80% of which are pyramidal cells and 20% are interneurons (Braitenberg and Schüz, 1998). To mimic physiological measurements, in which a group of cells responds to a preferred stimulus while the rest are indifferent, each stimulus activates a small and distinct subpopulation of *f N*_*E*_ excitatory cells (*f* = 0.15). The remaining (1 - 2*f*) *N*_*E*_ neurons did not respond to either of the stimuli. The network encodes only two directions of stimuli (left or right) and uses a small subpopulation of neurons to respond to each stimulus. Simulations were performed with *N*_*E*_ = 384 and *N*_*I*_ = 96.

#### 2.5.1. Neurons

Specific properties of neurons can be defined in the model so that distinctive properties of neurons, such as RMPs and membrane capacitance, found in experiments, can be applied to study how they can affect neural activities differently under identical given conditions.

Both pyramidal cells and interneurons in the model are represented as leaky integrate-and-fire neurons (Tuckwell, 1988). They are characterized by a resting potential of *V*_*L*_ = −70 *mV*, a firing threshold of *V*_*th*_ = −50 *mV*, a reset potential of *V*_*reset*_ = −60 *mV*, a membrane capacitance of *C*_*m*_ = 0.5 *nF* for pyramidal cells and *C*_*m*_ = 0.2 *nF* for interneurons, a membrane leak conductance of *g*_*L*_ = 25 *nS* for pyramidal cells and 20 *nS* for interneurons, and a refractory period of τ_*ref*_ = 2 *ms* for pyramidal cells and 1 *ms* for interneurons. The corresponding membrane time constants were $\tau_{r\,e\,f}=\frac{C_{m}}{g_{L}}=20\,m\,s$ for excitatory cells and 10 *ms* for interneurons, as reported by McCormick et al. (1985). When the membrane potential of a cell is below the threshold, the membrane potential *V*(*t*),


$$C_{m}\,\frac{d\,V\,\left(t\right)}{d\,t}=-g_{L}\,\left(V\,\left(t\right)-V_{L}\right)-I_{s\,y\,n}\,\left(t\right),$$

The rate of membrane potential change differs based on the difference between the current *V*(*t*) and *V_L_* to which the potential naturally returns when there are no synaptic inputs. The total current flowing into the cell owing to all synapses is represented as *I*_*syn*_ (*t*).

#### 2.5.2. Synapses

The network comprises connections between pyramidal cells and interneurons (Figure 2A), which receive recurrent EPSCs via AMPA and NMDA receptors. It receives inputs from two sources: right and left and background noise. NMDA receptors receive inputs only from the local network, whereas AMPA receptors receive external inputs.


$$I_{s\,y\,n}\,(t)=\,I_{e\,x\,t,A\,M\,P\,A}\,\left(t\right)+I_{r\,e\,c,A\,M\,P\,A}\,\left(t\right)+I_{r\,e\,c,N\,M\,D\,A}\,\left(t\right)$$

$$+I_{r\,e\,c,G\,A\,B\,A}\,\left(t\right)$$

where


$$I_{e\,x\,t,A\,M\,P\,A}\,\left(t\right)=g_{e\,x\,t,A\,M\,P\,A}\,\left(V\,\left(t\right)-V_{E}\right)\,s^{e\,x\,t,A\,M\,P\,A}\,\left(t\right)$$

$$I_{r\,e\,c,A\,M\,P\,A}\,\left(t\right)=g_{e\,x\,t,A\,M\,P\,A}\,\left(V\,\left(t\right)-V_{E}\right)\,\sum_{j=1}^{C_{1}}w_{j}\,s_{j}^{A\,M\,P\,A}\,\left(t\right)$$

$$I_{r\,e\,c,N\,M\,D\,A}\,\left(t\right)=\frac{g_{N\,M\,D\,A}\,\left(V\,\left(t\right)-V_{E}\right)}{(1+\left[Mg^{2+}\right]\text{exp}\left(-\frac{0.062\,V\,\left(t\right)}{3.57}\right)}\,\sum_{j=1}^{C_{E}}w_{j}\,s_{j}^{N\,M\,D\,A}\,\left(t\right)$$

$$I_{r\,e\,c,G\,A\,B\,A}\,\left(t\right)=g_{G\,A\,B\,A}\,\left(V\,\left(t\right)-V_{I}\right)\,\sum_{j=1}^{C_{1}}s_{j}^{G\,A\,B\,A}\,\left(t\right)$$

The *V*_*E*_ and *V*_*I*_ represent the membrane potentials (MPs), with *V*_*E*_ = 0 *mV* and *V*_*I*_ = −70 *mV*. The dimensionless weight *w_j_* represents the structured excitatory recurrent connections. The sum over *j* represents the sum of all synapses connected to the presynaptic neuron *j* in the local network. In this model, the NMDA currents were dependent on the extracellular magnesium concentration, [*Mg*^2 +^] = 11 *mM*.

The timescales of the different synaptic inputs are described as follows. The variables that control the opening and closing of channels—known as gating variables (s)—are described as follows: The AMPA channels, both external and recurrent, are described by


$$\frac{d\,s_{j}^{A\,M\,P\,A}\,\left(t\right)}{d\,t}=-\frac{s_{j}^{A\,M\,P\,A}\,\left(t\right)}{\tau_{A\,M\,P\,A}}+\sum_{k}\delta \,\left(t-t_{j}^{k}\right)$$

The time required for AMPA currents to decrease is represented as τ_*AMPA*_ = 2 *ms* (Hestrin et al., 1990; Spruston et al., 1995). The sum of *k* represents the sum of spikes emitted by presynaptic neuron *j*. In the case of external AMPA currents, the spikes are emitted according to a Poisson process with a rate of ϑ_*ext*_ = 2.4 kHz independently for each cell. NMDA channels are described by


$$\frac{d\,s_{j}^{N\,M\,D\,A}\,\left(t\right)}{d\,t}=-\frac{s_{j}^{N\,M\,D\,A}\,\left(t\right)}{\tau_{N\,M\,D\,A,d\,e\,c\,a\,y}}+\alpha \,x_{j}\,\left(t\right)\,\left(1-s_{j}^{N\,M\,D\,A}\,\left(t\right)\right)$$

$$\frac{d\,x_{j}\,\left(t\right)}{d\,t}=-\frac{x_{j}\,\left(t\right)}{\tau_{N\,M\,D\,A,r\,i\,s\,e}}+\sum_{k}\delta \,\left(t-t_{j}^{k}\right)$$

where the decay time of the NMDA currents is set to τ_*NMDA*,*decay*_ = 100 *ms*, the rise time constant is set to τ_*NMDA*,*rise*_ = 2 *ms* and the amplitude of the NMDA current is set to α = 0.5 *ms*^−1^. The equation governing the GABA synaptic variable can be expressed as follows


$$\frac{d\,s_{j}^{G\,A\,B\,A}\,\left(t\right)}{d\,t}=-\frac{s_{j}^{G\,A\,B\,A}\,\left(t\right)}{\tau_{G\,A\,B\,A}}+\sum_{k}\delta \,\left(t-t_{j}^{k}\right)$$

The decay time constant of GABA currents is represented as τ_GABA_ = 5 ms (Salin and Prince, 1996; Xiang et al., 1998). All synapses had a latency of 5 ms. In the *N* = 480 neuron network, the following values were used for the recurrent synaptic conductance (in *nS*): for pyramidal cells, g_ext,AMPA_ = 2.1, g_rec,AMPA_ = 0.5, g_NMDA_ = 0.165, and g_GABA_ = 1.3; for interneurons, g_ext,AMPA_ = 1.62, g_rec,AMPA_ = 0.04, g_NMDA_ = 0.13 and g_GABA_ = 1.0. These synaptic conductances are roughly similar to those measured in experiments (Destexhe et al., 1998). Three features are noteworthy: First, recurrent excitation is mostly mediated by NMDA receptors (Wang, 1999, 2001); second, the network is overall dominated by recurrent inhibition (Amit and Brunel, 1997; Brunel and Wang, 2001); third, neurons receive a large amount of stochastic background inputs.

#### 2.5.3. Structure of recurrent excitatory connections between pyramidal cells

Every neuron receives inputs from all the other neurons, but the strength of these connections is organized in a specific manner. Because of the Hebbian rule, which states that neurons that fire together frequently are more likely to have stronger connections than other neurons, groups of neurons that respond to similar external sensory inputs are likely to have “potentiated” synapses. Therefore, we set the strength of the neurons in the same group as *w*_*j*_ = *w*_+_ > 1 compared to the baseline with a synaptic strength of *w*_*j*_ = 1. These neurons tend to be involved in the making of similar decisions. Unless otherwise stated, we used *w*_+_ = 1.9. We varied the value of *w*_+_ to study the effects of recurrent synaptic strength. The strength of synaptic “depression” between two selective populations, and from a nonselective population to a selective one, is represented by a value of *w*_−_, which is less than 1. By contrast, the connections between other populations have a value of *w_j_* equal to 1. In the model, the overall recurrent excitatory synaptic drive in the spontaneous state remains constant as *w*_+_ is varied (Amit and Brunel, 1997) by setting


$$w_{-}=1-\frac{f\,\left(w_{+}-1\right)}{\left(1-f\right)}$$

Additionally, synaptic efficacy remained fixed throughout the simulation, assuming that the overall influence of recurrent excitatory synapses during spontaneous activity remained unchanged when the value of *w*_+_ was altered (Amit and Brunel, 1997). The strengths of the synapses were maintained constant throughout the simulation. The population firing rates *r_A_* and *r_B_* were calculated by counting the total number of spikes in each of the two neural groups during a time window of 20 *ms*. The spike count was then divided by the number of neurons and the length of the time window.

#### 2.5.4. Simulations

Computer-based simulations were performed on a MacBook Pro workstation, utilizing a customized version of the RK2 method [as described in Hansel et al. (1998) and Shelley and Tao (2001)] for the numerical calculation of the equations that govern the behavior of all cells and synapses. The time increment used in the integration process was set as 0.02 *ms*. The computer simulation was conducted using Python code, which is freely available online^1^ (Wong and Wang, 2006; Gerstner et al., 2014).

## 3. Results

**FIGURE 1:**
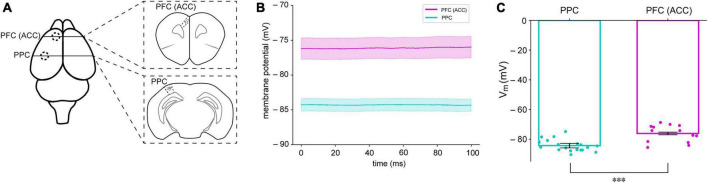
Different membrane potentials (MPs) in the PFC and PPC of mice. **(A)** The PFC and PPC regions where brain slices were obtained for whole-cell recording **(B)** Averaged resting membrane potentials (RMPs) of neurons in the PFC (magenta) and PPC (cyan) in mice. The shaded areas indicate standard error of mean (SEM) **(C)** Summary of RMPs in PPC (*n* = 19) and PFC (*n* = 13). ***P < 0.05.

The resting membrane potential (RMP) levels in the brain are influenced not only by the behavioral state of the animal, but also by the specific layer within a cortical column (Crochet and Petersen, 2006; Crochet et al., 2011; Poulet et al., 2012; Eggermann et al., 2014). To investigate the possibility of these differences in RMPs in areas related to cognitive tasks, we performed whole-cell recording in the area of prefrontal cortex (*n* = 13, 1.0 AP, ±0.5 ML from bregma, −0.5 DV from the brain surface) and posterior parietal cortical area (*n* = 19, −2.0 AP, ±1.5 ML from bregma, −0.5 DV from the brain surface) in mice. We measured the MPs of neurons in these areas. In the present study, the levels of membrane potential of PFC were generally 10 mV higher than those of PPC (PPC = 84.29 ± 1.52 mV, PFC = 76.12 ± 0.89 mV, *p* = 0.001) (Figure 1). Except the whole cell capacitance our experimental findings revealed notable distinctions between the cells of prefrontal cortex (PFC) neurons and posterior parietal cortex (PPC) neurons. While both regions displayed similar values of the whole cell capacitance, with PPC neurons at 39.61 ± 3.23 pF and PFC neurons at 40.05 ± 2.96 pF, the rheobase values for PFC neurons were lower compared to those of PPC neurons (115.625 ± 16.90 pA and 213.15 ± 13.70 pA, respectively). Also, the voltage changes required to trigger action potentials were also markedly different between the two regions (47.85 ± 1.75 mV for PPC and 36.87 ± 1.83 mV for PFC), and input resistance was found to be higher in PFC neurons (97.92 ± 6.47 MΩ) compared to PPC neurons (72.53 ± 3.96 MΩ).

**FIGURE 2:**
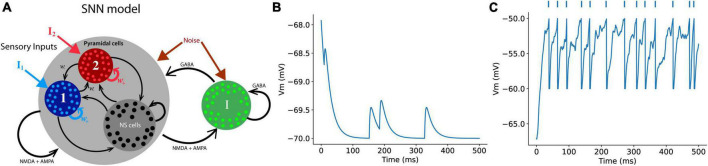
Schematics of a spiking neural network (SNN) **(A)** The SNN model in this study contains a total of 384 excitatory (pyramidal cells) and 96 inhibitory neurons. Each group of a chosen type comprises 96 neurons that specifically responds either right or left external sensory inputs, and the rest of excitatory neurons are non-specific (NS cells) that have no preferred choice type. Each neuron in the model receives three types of synaptic inputs (AMPA, GABA, and NMDA) **(B)** An example of the RMPs of a neuron that receives AMPA inputs from presynaptic neurons in the network. **(C)** When the post-synaptic neurons receive enough number of synaptic inputs, the neuron discharges an action potential, the RMP returns to the reset voltage set in the model (–60 mV in this case). The dots on the top of the plot represent spike events.

We used a SNN introduced in the decision-making process (Wang, 2002). The most distinctive characteristic of this model is that we can introduce synaptic transmission dynamics into the model and monitor their behavior during the decision-making process. This model successfully describes and mimics single-cell neural activities during cognitive tasks related to working and decision making (Goldman-Rakic, 1995; Shadlen and Newsome, 1996). This model assumes two types of sensory information (external inputs to the network). Once a specific type of information for decision-making reaches a specific group of related population in the neural network, neurons in the group starts increasing their firing rates while exciting the neurons in the same group through recurrent synaptic connections among neurons in the group. This leads to the winner-take-all phenomenon (Wang, 2002), in which a group of neurons related to a type of choice keeps increasing their firing rates, while the other group of pyramidal neurons for the other alternative choice suppresses activities through a group of inhibitory neurons that are connected to both groups of pyramidal neurons (Figure 2). A theoretical study reported that the most distinct difference between the PPC and PFC is recurrent synaptic structure (Murray et al., 2017). This difference in the level of connections of recurrent structure that connect neurons in the same group for a type of decision was solely able to explain the specific neuronal network dynamics shown in the experimental studies. They were able to show that stronger recurrent structure causes a faster increase in the population firing rate of a neural network, indicating that the population firing rate of the neural network in the PFC with higher recurrent synaptic strengths increases faster than that in the PPC, which possesses lower synaptic strengths among neurons with the same type of preferred decision cues.

**FIGURE 3:**
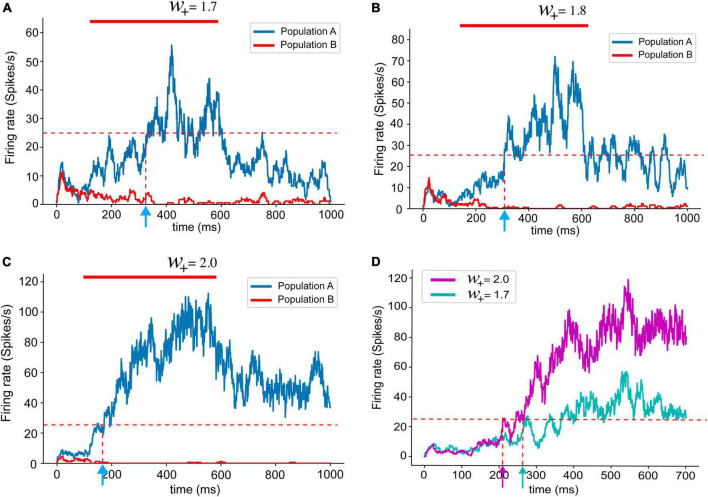
Population firing rates with different recurrent synaptic strengths **(A–C)**. The red bar marks stimulus presentation. As introduced in the previous study (Murray et al., 2017), the population firing rates obtained from spiking neural network models shows shorter reaction times with stronger recurrent synaptic connections. **(D)** Overlapped population firing rates of networks with *w*_+_ 2.0 (magenta) and *w*_+_ = 1.7 (cyan). Stronger recurrent synaptic connections yield higher population firing rate and shorter reaction time.

As suggested by Murray et al. (2017), a neural network with stronger recurrent synapses showed faster ramping up of population dynamics (Figure 3). When *w*_+_is lower than 1.7, the neural network was not able to hold persistent activities, which is an essential feature for the PPC or PFC for required cognitive functions, such as working memory. Therefore, we set a range of values for *w*_+_for the analysis in this study from 1.7 to 2.2 (Supplementary Figure 1). The value of 2.2 is the limit at which the network shows the highest reasonable population firing rate, as shown in the experimental data. In previous experiments, firing rates generally did not exceed 100 spikes/s (Shadlen and Newsome, 1996, 2001; Roitman and Shadlen, 2002).

**FIGURE 4:**
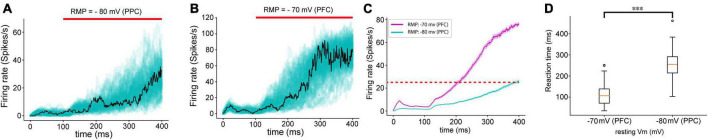
Neural network dynamics (reaction time) changed by different levels of MPs. **(A)** Overlapped population firing rates of 100 trials with the RMPs found in PPC (–80 mV) and **(B)** in PFC (–70 mV). The red bar indicates external sensory input stimulation applied. The solid black line represents the population firing rate with a median reaction time value. **(C)** Averaged population rate (solid lines) of 100 trials with SEM in shaded area. **(D)** Boxplot of reaction time during which the population firing rate of the neural network reaches 25 Hz from the stimulation onset. The population firing patterns of the neural networks with lower (PPC) and higher (PFC) MPs set with values found in our experiment resemble the firing patterns revealed by the experiment data and the results introduced in a previous study (Murray et al., 2017). ***P < 0.05.

Because we found distinctively different levels of base membrane potential, we applied the values found in our experiment to the neural network model for the decision-making process (Figures 4A, B). We chose the RMP as a primary variable for our modeling parameters because it exhibited both significant differences between the PPC and PFC and offered a straightforward interpretation while monitoring the modeling results. To our surprise, altering the RMP had a notable impact on the behaviors of the neural network. The neural network with a lower base membrane potential showed longer durations of ramping than the network with a higher base membrane potential (Figures 4C, D). This finding emphasizes the influential role of RMP in shaping the dynamics and functioning of the modeled neural system.

**FIGURE 5:**
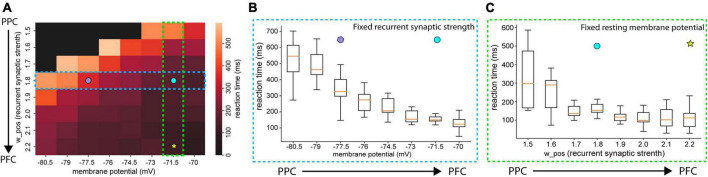
Averaged reaction time with various RMPs and recurrent synaptic strengths **(A)** heatmap of reactions times. The color of each cell indicates the averaged reaction time of 10 trials. The black-colored cell indicates that the population firing rate of the neural network did not pass 25 Hz in any of those 10 trials. **(B)** Boxplot of reaction time as RMPs set in the neural networks increase with the fixed recurrent synaptic strength (*w*_+_ 1.8). **(C)** Box plot of the reaction time as recurrent synaptic strength set in the neural networks increase with the resting membrane potential (RMP = –71.5 mV). The difference in reaction times between the networks of RMP = –77.5 mV (purple polygon) and RMP = –71.5 mV (cyan circle) in **(B)** is greater than the one between *w*_+_ = 1.8 (cyan circle) and *w*_+_ 2.2 (yellow star).

We subsequently investigated how reaction time is affected by either recurrent synaptic strength or RMPs levels (Figure 5A). As we studied in Figure 3, the reasonable range of synaptic strength from *w*_+_ = 1.7–2.2, since persistent activities during the delay period start appearing when the recurrent structure value is set to *w*_+_ = 1.7. At *w*_+_ = 2.2, the firing rate of the population was close to 100 spikes/s. A firing rate of 100 spikes/s is considered biologically feasible. The difference between the reaction time resulted from the neural networks with the values of *w*_+_ = 1.7 and *w*_+_ = 2.2 (40 *ms*) is less significant than the difference in the reactions times of the neural networks with different resting membrane potential levels between RMP = 77.5 *mV* and 71.5 *mV* (180 *ms*) with the same recurrent synaptic strengths *w*_+_ = 1.8 (Figures 5B, C). Therefore, we can conclude that for neural networks to have more distinctive functions through neural network dynamics, such as more PPC-like or PFC-like functions, the differences in the membrane potential can be critical.

**FIGURE 6:**
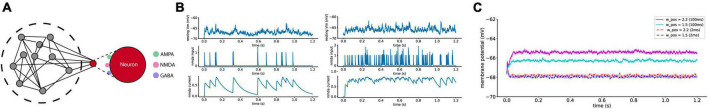
Single-cell modeling to mimic the whole-cell recording session. **(A)** The schematic of single-cell model with three major synaptic inputs of AMPA, GABA, and NMDA. **(B)** The effect of NMDA synaptic inputs to the RMP with lower (left) and higher (right) frequency. One of the most distinctive features of NMDA synaptic inputs is that the level of synaptic current saturate once enough number of synaptic inputs occur. **(C)** In this model, the mean frequency and strength of the NMDA synaptic inputs remained constant, while the time constants of synaptic transmission were varied. Increasing the strength of recurrent synapses led to an increase in (RMP) in the recipient neuron, but this effect was observed only with slow NMDA synaptic transmission inputs (100 *ms*) and not with fast inputs (20 *ms*). Averaged RMPs (solid lines) of 100 trials with the SEM in the shaded area.

To track the mechanism of higher membrane potential values, we built a single neuron model with various types of synaptic inputs to a single cell (Figure 6A). As a neuron in a noisy neural network constantly receives synaptic inputs from other neurons in the network, the single neuron in this model receives 1,000 excitatory and 250 inhibitory synaptic inputs that fire randomly following the Poisson process with a mean firing rate of 10 Hz. In this model, the mean frequency and strength of the NMDA synaptic inputs were fixed remained constant while the time constants of the synaptic transmission were varied to examine the impact of slow NMDA inputs to the MPs of the single neuron. This single neuron exhibits three types of synaptic transmission: AMPA, GABA, and NMDA, with different synaptic time scales. Previous studies have reported that slow synaptic transmission in the PPC and PFC areas is an essential factor that induces persistent working memory (Tsukada et al., 2005). It has also been suggested that NMDA receptor be the main source of this slow synaptic current to neurons in the PPC and PFC (Wang, 1999, 2002). Hence, we built a single neuron model to control the amount of NMDA synaptic inputs to the neuron by changing the synaptic strengths of the NMDA inputs. One of the most distinctive characteristics of synaptic transmission mediated by NMDA receptors is summations and saturation (Hestrin et al., 1990). The single-cell model shows that NMDAR-mediated EPSCs with a longer time scale saturate once a sufficient number of incoming presynaptic spikes arrive (Figure 6B). Figures 6B, C show the lifted RMPs with a higher NMDA input to the post-synaptic neuron. In Figure 6C, the time constant of NMDAR-mediated EPSCs was shortened from 100 *ms* (magenta and cyan) to 2 *ms* (red and blue). RMP levels significantly differ according to recurrent synaptic strength at 100 *ms* of the time constant of NMDAR-mediated EPSCs. The difference in the levels of RMPs, however, with 2 *ms* of the time scale of incoming EPSCs (no NMDAR-mediated EPSCs) was visibly reduced. It appears that an increase in the strength of recurrent synaptic inputs does not lead to a membrane potential increase if the synaptic time constant is short (e.g., 2 *ms*, as in the case of AMPA). These findings suggest that the slow synaptic input current, likely mediated by NMDA receptors and characterized by a longer synaptic time constant of approximately 100 *ms*, is the primary factor responsible for the increase in RMPs. Taken together, the modeling results reveal a highly possible mechanism of higher membrane potential levels in a higher cortical area, the PFC.

## 4. Discussion

In this study, we introduced the importance of elevated RMPs based on new observations of different levels of RMPs in the PFC and PPC. From our experimental data of whole-cell recording, we tracked a possible mechanism for the lifted membrane potential in the PFC. To our knowledge, this is the first study to report that membrane potential levels are generally different in the areas of PPC and PFC. These two areas have been studied with respect to decision-making and working memory, and the mechanistic processes at the circuit level have also been widely studied. Based on this knowledge, we investigated how these physiological differences affect the neural network dynamics that elicit the cognitive functions generated in these two areas.

We showed that the different levels of MPs of neurons in the network can affect neuronal network dynamics, eliciting desired cognitive functions (slow and fast ramping up dynamics) in the PPC and PFC, respectively. When the MP levels were set to the value that we found in our whole-cell recording experiment, the network showed similar population firing patterns in the PPC and PFC, as shown in *in vivo* experiments (Hanks et al., 2015). Our study is an example of using experimental data, theoretical background knowledge, and simulation results acquired from a neural network model to investigate the reasons for physiological phenomena and how these phenomena affect neural activities at the network level. A theoretical study has suggested that distinct recurrent synaptic structures in the neural networks of the PPC and PFC give rise to unique population dynamics that are essential for specific cognitive behaviors (Murray et al., 2017). Since the previous study used a population firing rate-based model, there were limitations in monitoring single-cell properties in the population that can be altered by synaptic strengths, including the RMP levels. The novelty of this study lies in the application of the RMPs values found in our experiment to the neuronal network model. Population firing rates acquired from our model simulations applying RMPs levels of neurons in the PPC and PFC showed similar patterns of population firing rates to those introduced in the theoretical study, indicating the possibility that stronger recurrent synaptic strengths in the PFC might have contributed to the elevated RMPs of neurons in that area.

Using the modeling toolkit, it is possible to modify various synaptic properties to study how new experimental findings can affect neural network dynamics, thereby elucidating the link between animal behaviors and neural network dynamics altered by changes in synaptic functional properties.

In the present study, we introduced a novel method using an available toolkit for neural network modeling. We applied the detailed parameters found in our experiments. This approach may enable researchers to investigate how functional modifications in synaptic transmission can affect neural dynamics, resulting in notable changes in animal behavior.

### 4.1. Reason for the lifted RMP: NMDA receptors as a contributor for the lifted resting membrane potential in the PFC area

Generally, RMP is closely related to excitability of neurons since it establishes the amount of input current required to reach the threshold for generating an action potential. The importance of appropriate levels of membrane potential in different regions for various functions has been widely studied (Kadir et al., 2018). RMP levels can be influenced by several factors, including the activity of specific ion channels, such as HCN and Kv4.2 (Hoffman et al., 1997; George et al., 2009; Eggermann et al., 2014). Factors such as the distribution of ion channels or pumps embedded in their cell membranes, as well as physical properties including membrane and axial resistance and membrane capacitance, could potentially contribute to these variations in RMP. For example, our observations revealed that the input resistance of neurons in the PFC was significantly higher compared to those in the PPC. This difference in input resistance may have played a role in the higher RMPs observed in the PFC.

Neuromodulators also alter RMPs, affecting cognitive states such as attention, arousal, and stress response by changing the responsiveness of a neuron to synaptic inputs. Noradrenaline and serotonin, for example, increase inward current to counteract the hyperpolarizing effects of potassium currents to maintain a neuron’s RMP for generating action potentials with appropriate synaptic inputs (Pape and McCormick, 1989). In our study, however, the RMP levels were measured in the brain slices of the PPC and PFC areas during a resting state, without the presence of specific input neuromodulators or apparent stimulation. Thus, the influence of other factors, such as specific ion channels or neuromodulators, on the measured RMP levels was likely minimal. Instead, we hypothesized that the distinct morphological differences between the PPC and PFC areas, specifically in the basal dendrites of pyramidal cells, may provide a more plausible explanation for the observed variations in RMPs (González-Burgos et al., 2019).

González-Burgos et al. (2019) has found that in comparison to the posterior parietal cortex (PPC), the prefrontal cortex (PFC) exhibited significantly larger basal dendrites, with a total length approximately 54% longer and a convex hull volume approximately 43% larger. According to their study, the researchers found that the total number of basal dendritic spines per pyramidal neuron was estimated to be 89% higher in the PFC compared to the PPC, based on mean spine density and total basal dendrite length. These cortex basal dendrites, especially in rodents, display NMDA spikes that are known to contribute recurrent excitation (Markram et al., 1997; Lisman et al., 1998; Nevian et al., 2007; Gökçe et al., 2016). These studies suggest that the stronger recurrent connections observed in the prefrontal cortex (PFC) could involve NMDA spike-mediated connections. The NMDA spikes may have contributed to higher resting membrane potential. In our study, we tested the hypothesis that synaptic time scales can affect the resting membrane potential (RMP) of neurons by comparing the changes in RMPs of two neurons only with different time scales (100 *ms* for NMDA-like and 2 *ms* for AMPA-like) in a setup where neurons receive inputs stochastically from presynaptic neurons, as in the brain slice experiment setup.

In a theoretical study, Murray et al. (2017) suggested that the recurrent structure is a key element that induces differences in neural network dynamics for optimal performance in the PPC and PFC. However, the study did not identify the components of the recurrent structure that may have contributed the most to distinctive neural network dynamics. Our study suggests that a possible component of the recurrent structure is NMDAR-mediated synaptic transmission, especially with a longer time constant.

Our modeling simulations showed that slow post-synaptic input through NMDA receptors can elevate RMP levels, while shorter synaptic transmissions, such as AMPA, have only transient impacts on the RMPs and affect them less. An anatomical study identified more NMDA synaptic connections in the PFC than in the PPC. González-Burgos et al. (2019) has found that there are more basal dendrites that receive NMDA spikes in the PFC than in the PPC in non-human primates. Based on their study, along with the simulation results in our study, the lifted level of membrane potential may have been due to the increased number of synaptic boutons in the PFC area rather than the number of AMPA receptors.

However, another study showed that the time constant of NMDAR-mediated synaptic transmission is more important than the number of NMDA receptors itself (Wang et al., 2008). This study found that even though the ratios of NMDA/AMPA receptors were similar in the PFC and V1 of rats, NMDAR-mediated synaptic transmission was much slower in the PFC than in V1 due to the higher expression of NR2B in the PFC. NR2B is also related to long-term potentiation and tends to decrease the extent of the expression as an animal ages (Cui et al., 2013). Although a comparison of NR2B expression in the PPC and PFC has not been elucidated, it is expected that NR2B might be expressed less in the PPC than in the PFC, based on the results of our experiment and modeling simulation. A study reported that inactivation of NMDA receptors in the PPC of rats did not have a major impact on the ability of the animals to perform working memory tasks (Goard et al., 2016; Scott et al., 2019). Taken together, it seems that more NMDAR-mediated synaptic transmissions in the PFC lift the RMPs of the PFC by slow synaptic transmission through NR2B receptors. Therefore, blocking NR2B receptors may reduce the RMPs levels.

Furthermore, there is extensive recognition that animals exhibiting schizophrenia (SZ) display impaired working memory (Eryilmaz et al., 2016). The dysfunction in maintaining population activities for working memory in SZ may be linked to NMDAR-mediated synaptic transmission, as suggested by the NMDAR hypofunction hypothesis (Amit et al., 1983). This hypothesis, developed in the 1980s, emerged from observations that administering NMDAR antagonists to healthy individuals could reproduce a broad range of positive, negative, and cognitive symptoms associated with schizophrenia (Krystal et al., 1994; Lee and Zhou, 2019). In the context of SZ models, this suggests that even with numerous incoming inputs from other neurons in the network, a deficient number of NMDA receptors can impede the adequate increase in RMPs necessary to sustain persistent activities for working memory. Moreover, variations in MPs in this study were observed in slice experiments. Conducting *in vivo* whole-cell recordings in the PFC and PPC during decision-making tasks will provide a deeper understanding of the relationship between different MP levels and distinct network dynamics in these regions.

### 4.2. Lifted membrane potential and neural network dynamics

According to the theoretical study mentioned above, the PPC with relatively weaker recurrent synaptic connections provides computational advantages when the neural network accumulates evidence by reducing the error rates in discrimination between choices, forming a weak network attractor. However, the PFC with a higher recurrent synaptic structure is more robust to distractors. Ramping up quickly in the population dynamics of the PFC area may have advantages in holding up choices (working memory), but changing the decision becomes difficult if a wrong decision is made. Therefore, slow ramping up in the PPC and a faster increase in the population firing rate in the PFC are suggested to be advantageous for performing cognitive tasks with fewer errors and more robustness to distractors.

Using the SNN model in this study, our simulation results verified that higher recurrent synaptic strength, as suggested by Murray et al. (2017), gives rise to more PFC-like neural dynamics, whereas weaker connections make the network behave more like the population neural activities in PPC. However, unlike the study that used the rate-model to describe neural behaviors (Murray et al., 2017), we used the SNN model to apply distinctive MP levels in the PPC (lower) and PFC (higher).

In addition, we showed that in a reasonable range of *w*_+_, which was determined by the general firing rates (∼50 spikes/s) shown in the network (Chafee and Goldman-Rakic, 1998), the levels of MP can affect neuronal network dynamics. Our study introduces the possibility that the lifted MP levels can be crucial in giving rise to distinctive neural network dynamics in the areas of the PPC and PFC, implying that the lifted MPs of neurons in these areas could not just be epiphenomenal, but necessary for each area to have optimized performance in cognitive tasks. For example, with the same number of synaptic transmissions of either AMPA or NMDA, more time for synaptic input integration is needed to reach an action potential threshold if the MP is lower. This delayed time may be the reason why neurons in the PPC gradually increase their firing rates while accumulating more evidence than neurons in the PFC. Likewise, the elevated RMPs of neurons in the PFC, inducing shorter distances from RMPs to the firing threshold, may enable them to encode incoming evidence more categorically than the PPC, since the same number of AMDAR- or NMDAR-mediated synaptic inputs will more promptly increase the firing rate of a single cell, which results in a rapid increase in the population firing rate of the PFC neural network. This relatively shorter time to generate action potentials may contribute to the physiological mechanism that helps the PFC network be more robust to distractions and more likely to induce errors in making decisions.

We propose that the lower and higher RMP levels in the PPC and PFC, respectively, may be a necessary condition for each area to give rise to desirable cognitive functions, such as gradual evidence collection and attentional saliency for PPC and robustness of persistent activities (working memory) and filtering of distractors for PFC. Therefore, it is reasonable to conclude that stronger recurrent structure introduced in the previous study (Murray et al., 2017), which our study suggests, are derived from a greater number of NMDA receptors with slow synaptic transmissions that lift the RMPs. The increased RMPs by NMDAR-mediated synaptic transmission may, in turn, contribute to a faster ramping up in the PFC area relative to the PPC area.

## Data availability statement

The original contributions presented in this study are included in this article/Supplementary material, further inquiries can be directed to the corresponding author.

## Ethics statement

The animal study was reviewed and approved by the Animal Experiment Ethics Committee (approval no. IACUC-20-00007) of the Korea Brain Research Institute (KBRI).

## Author contributions

MY carried out all the computer simulations and analysis of simulation data, and co-wrote the manuscript. Y-SY performed whole-cell recording. J-CR designed whole-cell recording and contributed to all aspects of this project in interactions with JC and MY. JC designed the research, worked with the other authors throughout the project, and co-wrote the manuscript. All authors contributed to the article and approved the submitted version.

## Footnotes

1
https://github.com/EPFL-LCN/neuronaldynamics-exercises
