# Supplementary material for: Different resting membrane potentials in posterior parietal cortex and prefrontal cortex in the view of recurrent synaptic strengths and neural network dynamics
Source: Front Cell Neurosci. 2023 Jul 13;17:1153970. doi: 10.3389/fncel.2023.1153970 (PMC10372347; doi:10.3389/fncel.2023.1153970)
Supplement: Supplementary file 1 [file Table_1.docx]

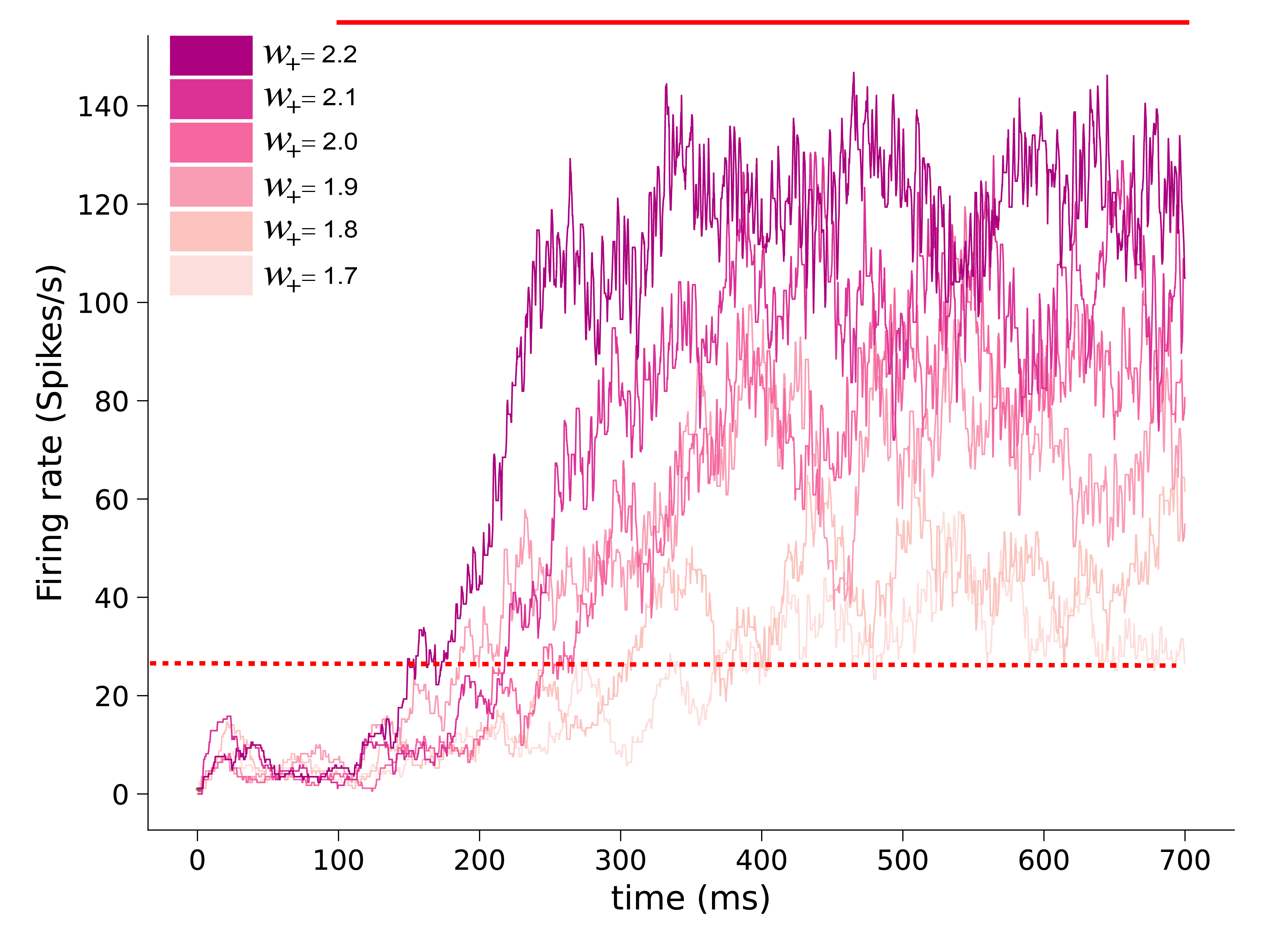


**Supplementary Fig 1**. Exemplar population firing rates with different recurrent synaptic strengths from $w_{+}$ = 1.7 to $w_{+}$ = 2.2. The red bar marks stimulus presentation. Higher synaptic connection strength results in increased population firing rates and reduced reaction times at which the firing rates surpass the threshold of 25 spikes per second.
